# Application of artificial intelligence in green building concept for energy auditing using drone technology under different environmental conditions

**DOI:** 10.1038/s41598-023-35245-x

**Published:** 2023-05-21

**Authors:** Osama Khan, Mohd Parvez, Monairah Alansari, Mohammad Farid, Yuvarajan Devarajan, Subash Thanappan

**Affiliations:** 1 Department of Mechanical Engineering, Jamia Millia Islamia University, New-Delhi, 110025 India; 2 Department of Mechanical Engineering, Al-Falah University, Haryana, 121004 India; 3 Department of Mathematics, King Abdulaziz University, Jeddah, Saudi Arabia; 4 Department of Mathematics, Deanship of Educational Services, Qassim University, Buraidah, 51452 Saudi Arabia; 5 Department of Thermal Engineering, Saveetha School of Engineering, SIMATS, Chennai, Tamil Nadu India; 6 Department of Civil Engineering, Ambo University, Ambo, Ethiopia

**Keywords:** Energy science and technology, Engineering

## Abstract

Thermal losses through weak building envelope is responsible for global current energy crises. Application of artificial intelligence and drone setups in green buildings can help in providing the sustainable solution the world is striving for years. The contemporary research incorporates a novel concept of measuring the wearing thermal resistances in the building envelope with the aid of a drone system. The above procedure conducts a throughout building analysis by considering three prime environmental parameters such as wind speed (WS), relative humidity (RH) and dry bulb temperature (DBT) with the aid of drone heat mapping procedure. The novelty of the study can be interpreted by the fact that prior researches have never explored the building envelope through a combination of drone and climatic conditions as variables in building areas difficult to access, thereby providing an easier, risk free, cost effective and efficient reading. Validation of the formula is authenticated by employing artificial intelligence-based software’s which are applied for data prediction and optimization. Artificial models are established to validate the variables for each output from the specified number of climatic inputs. The pareto-optimal conditions attained after analysis are 44.90% RH, 12.61 °C DBT and 5.20 km/h WS. The variables and thermal resistance were validated with response surface methodology method, thereby presenting lowest error rate and comprehensive R^2^ value, which are 0.547 and 0.97, respectively. Henceforth, employing drone-based technology in estimating building envelope discrepancies with the novel formula, yields consistent and effective assessment for development of green building, simultaneously reducing time and cost of the experimentation.

## Introduction

In recent times, energy requirements have increased considerably whereas the energy production resources have degraded significantly. This has led researchers to find alternative methods of preserving energy so as to meet the future potential demands. In India, the overall losses due to the temperature variations in building envelope is computed to be around 41% of the original energy requirements of the buildings (MOE). The present-day buildings across the world are subject to large energy losses which may primarily be due to irremovable circumstances^1,2^. These buildings are known to underperform on efficiency basis mainly in underdeveloped and developing countries, thereby differing from the original design of green building. According to a recent study^3^, approximately 63% of the energy being produced is procured by residential or industrial types of buildings. A broader data survey throughout India for the financial year 2018–2019 estimated the overall power produced by utilities to be approximately 1372 (Tera Watt-h)^4^. These utilities mainly comprise of indoor activities like coffee machines, microwave, heater etc.^5,6^. In developing countries, government have been taking forward steps to reduce these exergy losses by incorporating smart systems in the buildings^7^. This energy, if not properly utilized, results in substantial losses to the economy of a country and environment^8^.

The current need is to find effective ways to curb these losses and save the resources for a fruitful future. An effective way to check and monitor these losses is the evaluation of heat losses through building envelope^9,10^. The building envelope primarily comprises of all building setups such as walls, ceiling, windows, partitions, and doors. These fixtures are prime elements of heat losses in a building setup since the thermal energy transfusion happens throughout the day as the climatic conditions vary for 24 h^11,12^. These differences can be thought as the primary reasons of energy losses, henceforth lowering the overall efficiency of the building^13^. In some studies, these losses are directly related to building structures having weak overall outer coating^14^. These losses are an amalgamation of various building fixtures which may have inferior quality of insulation applied at the exterior of these fixtures. In addition, old structure tends to become weaker over the years resulting in substantial energy losses^15^. Furthermore, constant infiltration of outside air through various cracks and openings may also heighten the energy demand^16^. The above specified discrepancies can be sorted out by applying better quality materials at the start of the building, repairing existing designs, sealing up air gaps, and cracks^17^. Previous studies have used insulation to reduce building energy losses^18^, leading to better energy efficiency and trapping cooler air in summer and warmer air in winter. Simultaneously reducing thermal heat and moisture content inside rooms can lower energy requirements^19^. In past literatures, the specified heat transfer circuit is necessary to evaluate so as to contemplate the building load and energy losses within the building structure^20,21^. Often the building envelope is controlled by a single factor known as R-value, also stipulated as the thermal resistance^22^.

Prior literatures have seldomly explored the combination of drone monitoring under the influence of varying climatic conditions^23–25^. Halder and Afsari's^25^ systematic review in explores the use of robots for inspection and monitoring of buildings and infrastructure. Alkaabi et al.^26^ propose the use of drone-captured 3D thermal imaging to monitor the thermal environment of buildings and pedestrian spaces in order to contribute to the development of sustainable cities. Kopp et al.^27^ present a method for estimating radiative heat loss of building envelopes using 3D thermographic models created with small unmanned aerial systems (sUAS). Oh et al.^28^ propose a drone-assisted image processing scheme utilizing frame-based location identification for the detection of cracks and energy loss in building envelopes. Zheng et al.^29^ present a thermal performance detection method for building envelopes based on a 3D model generated by UAV thermal imagery.

Furthermore, authors did not find any article validifying the results of building monitoring through artificially intelligent developed model. It aids in identifying the major deficiencies within the building setup and further aids in development of proper energy saving concepts so as to enhance the overall working performance for any structural setup^30,31^. Moreover, using infrared technology in drone system in past have yielded effective results^32,33^.

The study aims to incorporate intelligent techniques for the development of a formula to accurately measure the wall temperature of a building with a drone. The research will focus on identifying the accuracy and reliability of using a drone to measure wall temperatures, as well as the limitations and challenges associated with this method. The study may also evaluate the feasibility of using drones for large-scale temperature measurements of high-rise buildings. The scope of the study will involve the development of a formula that incorporates intelligent techniques to accurately measure wall temperature, as well as the potential applications and recommendations for future research in this area. Although several studies have been directed in the field of AI and green building domain, but finding discrepancies in the building envelope through drones are still seldom and to the authors knowledge have never been explored so far. Novelty of the existing investigation is prevalent since absolutely no previous work has been reported for boosting building performance and reducing power usage to achieve green building while employing prediction models (ANFIS) from drone-based building audit while considering the environmental fluctuations. Earlier literatures have also shown the importance of soft computing techniques preferably ANN method to accurately predict building load in shorter time period with smaller dataset^34^. Furthermore, previous research have also employed response surface methodology (RSM) method since it is potent to produce a viable experimental test procedure even for a problem where data generation might be an issue especially in skyscrapers^35^.

In respect to information explained above, the researchers have come to a common deriving perspective as explained below:

- Employing drone-based systems in building to estimate achieve green building by finding the R-value demonstrates a practical and reasonable opportunity pertaining to its non-complex operation and social friendly cause.
- Investigation of thermal resistance for numerous environmental settings while engaging with intelligent software’s using a combination of ANFIS and RSM together have rarely been researched in any preceding work.
- Osama formula established for generating environmental variables yields datasets fairly adjacent to those attained while carrying out real time tests for green buildings.
- Preceding literatures (especially in the fields of thermal engineering) have emphasized the significance of uniting soft computing forecasting representations for building envelope power measuring, presenting precise R-value features through lower work applied, money applied, and man force applied^36,37^.

The subsequent sections comprise of data collection procedure and equipment’s required for the process.

## Materials and methods

The prime physics associated with the research is explained by interlinking the results achieved during experimentation is validified by the developed model. The prime theory of the research comprises of collecting data from building weak envelope with the aid of drone thermal images. Further these models are simulated in different climates. The reading are used to develop a formula. The validation of the formula is performed by AI models developed which show consistency in results as accuracy levels are high between experimental reading and developed model readings. Initial stage of functioning the operating procedures is performed by pre-explaining the inputs, followed by defining the outcomes of the study. The trial investigation is furnished by choosing environmental constraints which comprise of dry bulb temperature (DBT), wind speed (WS) and relative humidity (RH). For the proposed set of inputs, the thermal resistance and heat loss will be evaluated for the entire building envelope. To furnish the above criteria combination of prediction-optimization techniques have been employed which provides a comparative analysis between the investigational and prognosticated readings described in multiple consecutive phases: (a) Gathering datasets related with investigational data and clustering the datasets on the basis of training and testing sheets in separate excel file, (b) Model creation on the basis of environmental conditions hypothetical data generation through the new formula named Osama (c) Recognizing the finest performing model in artificial intelligence software for assessing the presentation of building structure, (d) Relative examination among the consequences of artificial intelligence, investigational and hypothetical frameworks for finest heat loss detection among them and (e) In conclusion, simplification and authentication of the outcomes with preceding representations.

**Figure 1:**
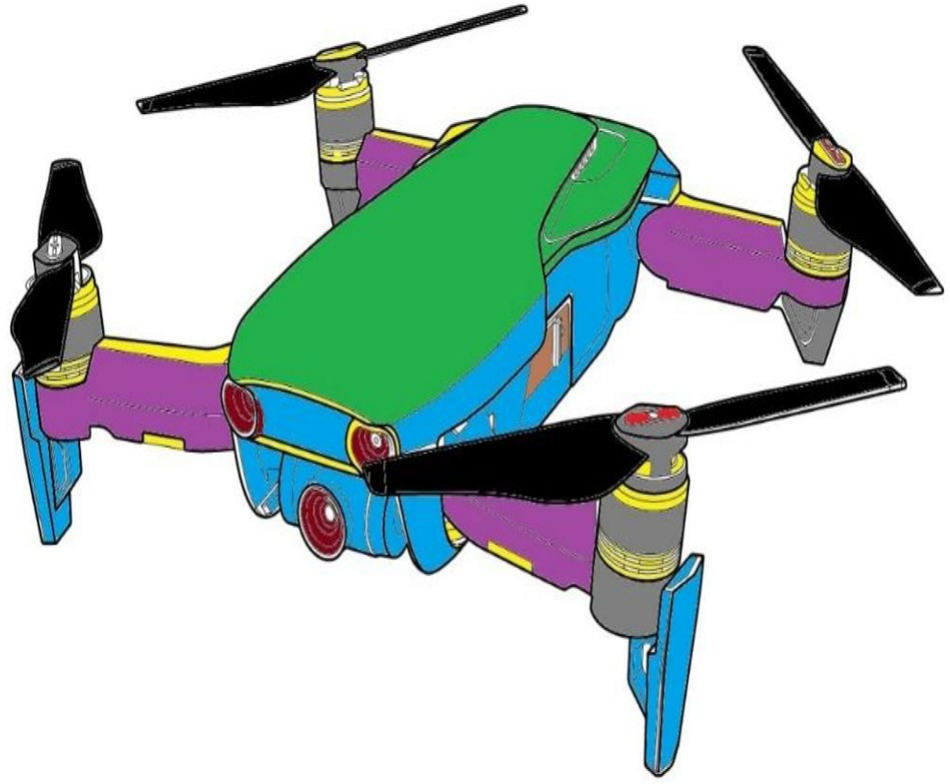
Different views of a Drone-camera arrangement.

**Figure 2:**
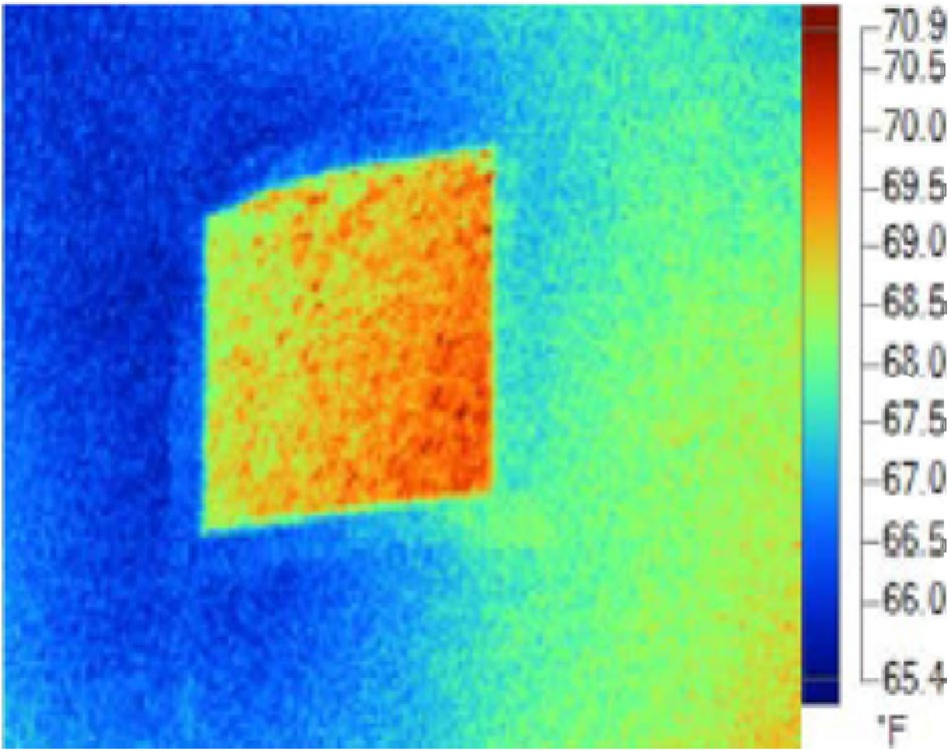
Crumbled aluminium foil.

The current research is mainly intended to evaluate the R-value of various fixtures in an existing building setup through experimental and theoretical estimations under different environmental conditions. The equipment setup employed in the study was a drone mounted infrared camera and portable infrared camera to contemplate the thermal resistance of all walls in an aging building setup. A Tello drone is integrated with a *Flir vue pro* camera capable of taking quick infrared pictures was used in combination with a portable camera thermal imaging for rapid data collection in this study. The three-dimensional view of the test drone attached with camera is displayed in Fig. 1. The analysis was performed on number of buildings in New Delhi. An aluminium foil was used which was initially crumbled and then made flat and later stuck on the test wall whose R-value need to be examined. The camera focussed a beam light towards the aluminium crumbled foil which detected the reflected temperature. The crumbled foil is shown in Fig. 2.

### Data compilation

The difference in energy requirement during the audit can be interpreted that building was designed for power usage on initial design which has deteriorated over the years and hence requires an audit to pinpoint exact location of losses. If this structure is correctly pinpointed and reinsulated, then it may save billions of rupees yearly. The investigational and theoretic information furnished for evolving the prognosticated model was generated from the UAV-IR arrangement. This setup technical rating is given below in Table 1.

**Table 1:** Details for drone camera arrangement.

S. No	Property	Specification
(A) Camera		Infrared Camera FLUE Meter
1	Angle of Viewing	23° × 17°
2	Heat accuracy	≤ 0.2 °C for 35 °C
3	Wavelength Level	8.2 to 20 μm
4	Indicator category	180 × 120 focal flat collection
5	IR camera	1020 × 960 resolution
6	Wall temperature Level	− 10 °C to 60 °C
7	Precision	± 0.2 °C or 0.2%
(B) Drone		PixHawk
1	Dimensions	9.8 × 9.3 × 4.1 cm
2	Weight	150 g
3	Battery Type	Lithium polymer
4	Flying Height	50 m
5	Flying Time	13 min

**Figure 3:**
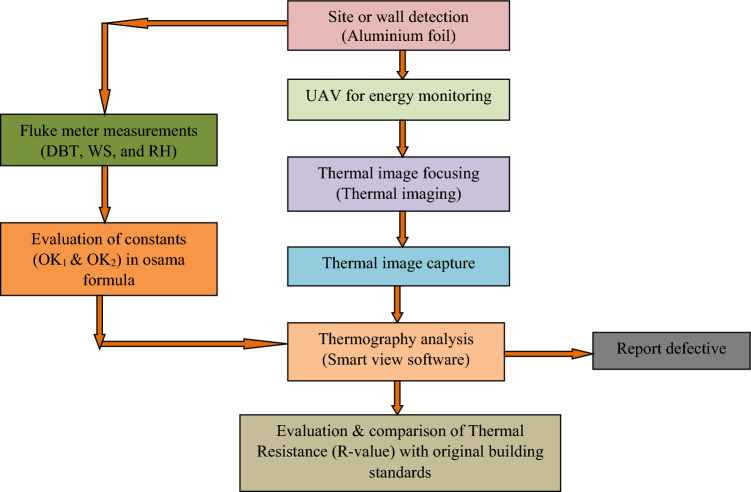
Flowchart for the working of drone-camera arrangement.

Data accumulation and experimentation is accomplished by the following flowchart displayed in Fig. 3. In the next section building specification is provided to understand the test area of the experiment.

### Building wall specifications in accordance with international standards

Infrared thermography technique is employed to stipulate the heat losses in the building setup in New Delhi, India. A Tello drone integrated with *Flir Vue pro* infrared camera is employed to attain information associated with building structures. The collected data was simulated with the help of software’s ‘SmartView’ and ‘*FLIR Reporter Pro’*. The present research was based on the standards specified by the ISO 6946:2007^38^, which is also known as the international standards specified for building envelope elements. The standard furnishes a few methods and strategies for evaluating the thermal resistance or R-value of all major building elements. The following assumptions for building were considered before testing:

- The building was constructed according to applicable building codes and standards.
- The building materials and components used in construction are of appropriate quality and durability.
- The building is not subject to significant environmental or natural hazards that could affect its structural integrity or safety.
- The building's occupants use the facilities in accordance with appropriate guidelines and regulations.

The method aids in obtaining the heat transfer rate through the building envelope elements. The primary reason of the R-value evaluation yields valuable input concerning building envelope which may require repairs through applying insulations at the pinpointed locations within the building. The research also highlights areas with insulation deterioration, heat leakages and energy losses associated with it. The research further highlights how these energy losses can be rectified with main focus on providing a cost-effective procedure. This enables the building to become energy efficient further providing a sustainable environment. The computed total R-value is displayed in Table 2. Subsequent section explains the development of the novel formula.

**Table 2:** Test walls original thermal resistances.

No	Surface specification	Width (m)	Thermal conductivity (W/mK)	Thermal resistance (m^2^K/W)
1	External surface	–		0.04
2	Outer leaf brick	0.103	0.78	0.132
3	Air gap	0.103		0.18
4	Concrete	0.204	0.78	0.23
5	Internal surface	–		0.13
	Total resistance			0.712

### Osama formula development

Conventional methods used to determine thermal resistance is not quite effective as it does not take in account the changes in environmental parameters (DBT, RH, and WS)^26^. The conventional formula for evaluating the thermal resistance without considering variation in ambient conditions is provided in Eq. (1):

1 $${R}_{th}=\left[\frac{\left({T}_{inside air} - {T}_{outside air} \right)}{\left[ {h}_{conv} ({T}_{outside air} - {T}_{inside wall})\right] + \left[ \varepsilon \sigma ({T}_{inside wall }^{4}- {T}_{outside reflected wall}^{4})\right]}\right]$$

The present study incorporates an integrated drone with infrared camera which captures thermal images which later must be transferred in software for R-value computation for tall rise buildings while taking environmental parameters in consideration. The primary reason of the R-value evaluation while considering environmental parameters yields valuable input concerning building envelope which may require repairs through applying insulations at the pinpointed locations within the building. previous studies have used similar formula but without considering the emissivity of the outside wall which is difficult to measure and access^27,28^. The current study obtains this temperature with ease by focussing thermos-graphic camera laser on aluminium foil and black tape attached on different locations of the building. These values are measured by variables O_K1_.

A novel formula Osama is developed to measure the thermal resistance of any wall with the aid of a drone keeping the ambient conditions in consideration. Furthermore, influence of input environment parameters is derived by interpolating the variation in the two variables (O_k2_) developed in the formula which previously was understated and never considered in earlier studies^29^. Environmental factors such as DBT, RH, WS, convective heat transfer coefficient (h) largely impact the temperature detection process, which further lead into contemplating the R-value. The variable O_k1_ and O_k2_ takes in consideration the effects of relative humidity, wind speed, and dry bulb temperature on thermal resistance. The formula was validated by testing its accuracy and predictive ability with ANFIS software by statistical tools RMSE and R^2^. The impact of the above parameters over the formula and the two constants was confirmed with a statical test known ANOVA to determine the significance level for each input environmental parameter. The formula provided below is used to compute the R-value for any wall in any environmental condition. This formula will aid in lowering the heat losses through wall by predicting the deteriorations in the building envelope as shown in Eq. (2):

2 $${R}_{th}=\left[\frac{\left({T}_{inside air} - {T}_{outside air} \right)}{\left[(1 / {O}_{{K}_{1}}) {h}_{conv} ({T}_{outside air} - {T}_{inside wall})\right] + \left[(1 / {O}_{{K}_{2}}) \varepsilon \sigma ({T}_{inside wall}^{4}- {T}_{outside reflected wall}^{4})\right]}\right]$$

where R_th_ is thermal resistance of the wall, T_inside air_ is the inside temperature of the building (probably maintained at 22 °C), T_outside air_ is the outside temperature or ambient temperature (also known as DBT), T_outside reflected wall_ is the temperature estimated with the camera after reflection from aluminium foil, h_conv_ is the convective heat transfer coefficient, ε is the emissivity, σ is the Stefan-Boltzmann constant, and O_k1_ and O_k2_ are Osama’s variables.

Primarily this model can be furnished as a global and flexible model applicable to all environments across the globe. This model is feasible since its cost-effective and efficient in energy losses in any building envelopes and replicates values obtained through experimentation. Present framework can be employed by researchers to estimate any savings achieved that can be used for building envelope element with an unknown R-value. Validation of the developed formula is explained in the next section.

### Modelling through artificial intelligence

**Figure 4:**
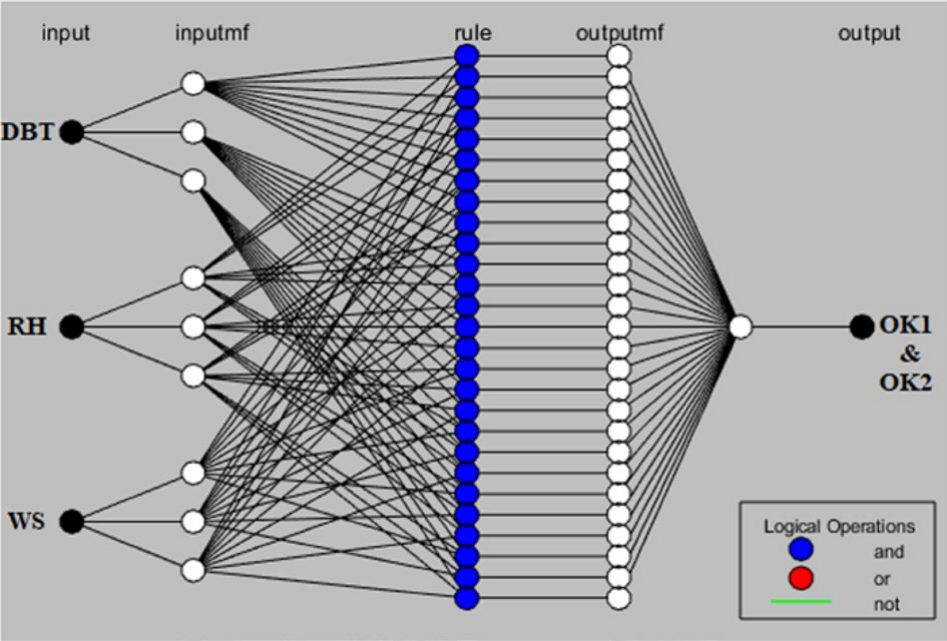
Framework of ANFIS Model.

The ANFIS model can be furnished by either employing Takagi–Sugeno framework and Mamdani framework. The present research has chosen the former method to obtain a feasible work as the number of inputs varied at different levels. Inputs were fed into the model and created the framework as evident from Fig. 4. Three models were created since the outputs were thermal resistance and their variables. The validity of the present research can be established by comparing the earlier engineering problems which too established the models on the present framework with utmost efficiency since these problems are so often limited, nonlinear, and uncertain database^39,40^. Recent applications pertaining to efficient results have paved for ANFIS popularity since it is a foremost tool in determining a feasible relationship among multiple inputs for numerous outputs. A Sugeno model consists of six major steps starting with the preliminary step of input constraints, trailed by fuzzification layer, rule consequent layer, rule strength normalization layer, rule consequent layer, and lastly the rule inference layer. Developing a viable algorithm facilitates the fuzzy theory and different member-ships being created by following a set of steps as explained between Eqs. (3)–(12). During experimentation thirty-two number of trial tests were furnished and were further divided into datasets one for training (24) while other for testing (8). The complete background explained for Sugeno algorithm is tabulated in Table 3.

**Table 3:** ANFIS framework for training the drone-camera based model.

Parameter	Value
Overall developed nodes	100
Amount of linear constraints	92
Amount of non-linear constraints	42
Amount of training information sets	56
Amount of fuzzy instructions	92

The above layers are explained empirically by applying ANFIS formulas to obtain final outcomes:

Step 1: Fuzzification step

3 $${Q}_{1,i}={\mu }_{{A}_{i}}(x) , \quad for \; i=\mathrm{1,2} or;$$

4 $${Q}_{1,j}={\mu }_{{B}_{j}}(y) , \quad for \; j=\mathrm{1,2};$$

5 $${\mu }_{{A}_{i}}(x) =\frac{1}{1+{\left[{\left(\frac{ x - {c}_{i}}{{a}_{i}}\right)}^{2}\right]}^{{b}_{i}}}$$

Step 2: Product step :

6 $${Q}_{2,i}= \underline{{w_{i} }} = {\mu }_{{A}_{i}}(x) {\mu }_{{B}_{i}}(y), \quad for \; i=\mathrm{1,2};$$

Step 3: Normalized step:

7 $${Q}_{3,i}=\underline{{w_{i} }} =\frac{{w}_{i}}{{w}_{1}+ {w}_{2}}, \quad for \; i= \mathrm{1,2}$$

Step 4: Defuzzied step:

8 $${Q}_{4,i}= \underline{{w_{i} }} {f}_{i} = \underline{{w_{i} }} ( {p}_{i}x + {q}_{i}y + {r}_{i}), \quad for \; i=\mathrm{1,2};$$

Step 5: Overall Yield step:

9 $$Q_{{5,i}} = overall\;output = \sum _{i} \underline{{w_{i} }} f_{i} = \frac{{\sum _{i} w_{i} f_{i} }}{{\sum _{i} w_{i} }}$$

10 $$f=\frac{{w}_{1}}{{w}_{1}+ {w}_{2}}{f}_{1}+\frac{{w}_{2}}{{w}_{1}+ {w}_{2}}{f}_{2}$$

11 $$f=\underline{{w_{1} }}( {p}_{1}x + {q}_{1}y + {r}_{1}) +\underline{{w_{2} }} ({p}_{2}x + {q}_{2}y + {r}_{2})$$

12 $$f = (\underline{{w_{1} }} p_{1} x + \underline{{w_{1} }} q_{1} y + \underline{{w_{1} }} r_{1} ) + (\underline{{w_{2} }} p_{2} x + \underline{{w_{2} }} q_{2} y + \underline{{w_{2} }} r_{2} )$$

Orthodox approaches applied in these types of complex problems require substantial time and skilled labour to develop the feasibility among input variables and final outcomes. Conversely, soft computing techniques are capable of furnishing a feasible inter relationship while simultaneously generating effective results without the obligation of any preceding datasets. Approximations and calculations generated from ANFIS procedure might be additionally fine-tuned by enhanced correctness and productivity by engagement of RSM method.

Frequently, the workability of ANFIS starts deteriorating for problems where the number of inputs become more than nine techniques since the outcomes might get trapped inside the local optima. Furthermore, the contradictory yields obscure the algorithm advancement. To overcome this complication, a hybrid formula named Osama formula is established capable of considering all the difficulties in the measurement process of climatic conditions while deriving composite building connected complications rapidly and effectually.

All major data applied and generated in the ANFIS models are provided in table. The discrepancy in the developed model could be explained with statistical tools such as coefficient of determination (R^2^) and mean-squared error (RMSE) provided in Eqs. (13) and (14), respectively.

13 $$RMSE=\sqrt{\frac{1}{N}{\sum }_{i=1}^{N}\Sigma {\left({P}_{i}-{E}_{i}\right)}^{2}}$$

14 $${R}^{2}=1-\frac{{\sum }_{i=1}^{N}\Sigma \left({P}_{i}-{E}_{i}\right)}{{\sum }_{i=1}^{N}\Sigma \left({P}_{i}-{E}_{m}\right)}$$

where $${E}_{m}=\frac{{\sum }_{i=1}^{N}\Sigma {P}_{i}}{N}$$

RMSE = Root Mean Square Error, R^2^ = Fraction of Variance, P_i_ = Forecast value obtained from modelling, E_i_ = Experimental value generated, E_m_ = Mean of the predicted values generated from models, N = Available Data, i = Trial run value need to be calculated.

### Response surface methodology

To a develop a feasible trial run set where input and output relationships can be obtained, we employ response surface methodology, which is capable of interlinking the numerous inputs with specified outcomes of the study. For this study, also, we interlinked the 32 trial runs with their respective fit equations. Moreover, the established relationship model specified the new extreme values for all input constraints. Drone modelling involves multiple set of inputs which were established from datasets validated from previous literature surveys that establish the viability of the experimental data and model development. For the pre-specified levels, the ranges developed were in strongly interrelated to the acquired experimental inputs, out of which the effects were non-beneficial to the end results. The climatic constraints comprised of dry bulb temperature which was ranged between 2 and 41 °C, relative humidity ranging between 20 and 80%, and wind speed in the range of 0–15 km/h. All trials were directed for diverse structure walls subjected to intricate geometrical dimensions for numerous climatic circumstances (DBT, RH, and WS) for the variables in the novel formula consequently to attain the finest combination among them based on outcomes. From the model interpretation different fits were developed fits equation for all outcomes and explained in the subsequent sections. The investigation comprises of numerous control factors, numerical and coded standards active in the convention planned Central Composite Rotatable Design collection (CCRD), covering all thirty-two trials. Comprehensive set of information under distinct climatic circumstances is presented in Appendix 1.

Vast domain has successfully implied RSM technique to furnish prognostic values with a faster and efficient way, while also simulating the problem according to its requirement. The tool also optimizes the responses based on the set of available parameters. Henceforth, RSM is often employed for performing simulations, optimization and vary levels of any number of inputs for a specific dataset. The investigated datasets are perceived with response surface regression method polynomial modelling of the second-order which were built by means of Eq. (15):

15 $$Y={\beta }_{o}+{\sum }_{i=1}^{k}1.{\beta }_{i}{X}_{i}+{\sum }_{i=1}^{k}2.{\beta }_{ii}{X}_{i}^{2}+{\sum }_{j\ge i}^{k}3.{\beta }_{ij}{X}_{i}{X}_{j}+ \varepsilon$$

where Y is the required outcome, X_i_ are numeric values of the factors, whereas terms β_0_, β_i_ , β_ii_ and β_ij_ are regression coefficients, i and j are linear and quadratic coefficients, and ε is the experimental error. Response surface graphs were drawn with the aid of these fitted representations.

### Evaluation of total thermal heat issued from the test walls

Several parameters are varied for analysing the thermal heat release from test walls. Primarily, the environmental parameters were altered with respect to various experimental readings for constants as outputs. Theoretically heat release rate (HRR) can be estimated with the following equations:

16 $$\frac{d{Q}_{G}}{d\theta }=\frac{d{Q}_{N}}{d\theta }+ \frac{d{Q}_{ht}}{d\theta }$$

where, dQ_G_/dӨ prescribes the total heat transfer proportion, dQ_N_ /dӨ the net heat transfer proportion, and dQ_ht_ /dӨ the heat transfer proportion to the walls.

To simplify analysis, the theoretical formula considers the air to behave as an ideal gas, where the term dQ_N_ /dӨ denotes the summation of the rate of work performed and the rate of variation of sensible internal energy within the room of a particular building. That is,

17 $$\frac{d{Q}_{N}}{d\theta }=P\frac{dV}{d\theta }+ \frac{dU}{d\theta }$$

18 $$\frac{d{Q}_{N}}{d\theta }=P\frac{dV}{d\theta }+ m{C}_{v}\frac{dT}{d\theta }$$

19 $$\frac{d{Q}_{N}}{d\theta }=\left(1+\frac{{c}_{v}}{R}\right)P\frac{dV}{d\theta }+ \left(\frac{{c}_{v}}{R}\right)V\frac{dp}{d\theta }$$

where, specific heat at constant volume is denoted by ${C}_{v}$ and specific heat ratio by $\gamma$.

Rejecting the temperature coefficient during differentiation leads the following equation:

20 $$\frac{d{Q}_{N}}{d\theta }=\left(\frac{\gamma }{\gamma -1}\right)P\frac{dV}{d\theta }+ \left(\frac{1}{\gamma -1}\right)V\frac{dp}{d\theta }$$

21 $$\frac{d{Q}_{ht}}{dx}=h{A}_{s}\left(T-{T}_{wall}\right)$$

Above equation comprises of a heat transfer coefficient (h), T_wall_ which denotes mean temperature attained in room walls, and A_s_ which is the surface area of wall^37^. Considering the fluid (air) undergoing turbulent flow, the equation by which can be estimated is given by the following formula:

22 $$h=\frac{a.\lambda }{C}{Re}^{0.7}+c\left(\frac{{T}^{4}-{T}_{wall}^{4}}{T-{T}_{wall}}\right)$$

where constant values are varied between, $0.35<a<0.8$, the usual Stefan-Boltzmann constant, $\lambda$ is the gas thermal conductivity and C is the wall coefficient. Uncertainties accounted is another aspect of an experimentation survey which is addressed in the next section.

### Uncertainty analysis

The investigation of error rates is evaluated through faults in the equivalent equipment’s. Inherent uncertainties are always associated in different set of instruments while measuring the parameters for input and output. The primary aim of this section is to reduce these errors and maximize the efficacy of the final outcome. Furthermore, research combined with uncertainty analysis section are deemed be accurate. The uncertainties might be available with different set of tools, measuring equipment, unskilled labour, and improper surrounding conditions. Therefore, to achieve a sense of reliability, all variables are measured thrice for every accessed walls.

In general, different techniques are used to estimate the investigational parameters (climatic) quantification and output parameters (O_k1_, O_k2_, and R-value) bring a minor error during experimentation. All the errors present in the equipment of the experimental analysis are prearranged in Table 4. The entire error rate is accessed in this experimentation by employing an empirical formulation established below:

23 $$U= {\left({\left[\frac{\partial R}{\partial {x}_{1}}{W}_{1}\right]}^{2}+{\left[\frac{\partial R}{\partial {x}_{2}}{W}_{2}\right]}^{2}+ \dots \dots . + {\left[\frac{\partial R}{\partial {x}_{n}}{W}_{n}\right]}^{2}\right)}^\frac{1}{2}$$

24 $${\text{The}}\;{\text{entire}}\;{\text{error}}\;{\text{rate}}\;\left( {\text{U}} \right) = {\text{square}}\;{\text{root}}\;{\text{of}}\;\left[ {\left( {{\text{error}}\;{\text{rate}}\;{\text{of}}\;{\text{DBT}}} \right)^{{2}} + \left( {{\text{error}}\;{\text{rate}}\;{\text{of}}\;{\text{WS}}} \right)^{{2}} + \left( {{\text{error}}\;{\text{rate}}\;{\text{of}}\;{\text{RH}}} \right)^{{2}} + \left( {{\text{error}}\;{\text{rate}}\;{\text{of}}\;{\text{reflected}}} \right)^{{2}} + \left( {{\text{error}}\;{\text{rate}}\;{\text{in}}\;{\text{estimation}}\;{\text{O}}_{{{\text{k1}}}} } \right)^{{2}} + \left( {{\text{error}}\;{\text{rate}}\;{\text{in}}\;{\text{estimation}}\;{\text{of}}\;{\text{O}}_{{{\text{k2}}}} } \right)^{{2}} + \left( {{\text{error}}\;{\text{rate}}\;{\text{in}}\;{\text{estimation}}\;{\text{of}}\;{\text{R}} - {\text{value}}} \right)^{{2}} } \right]^{{{1}/{2}}}$$

$${\text{The}}\;{\text{entire}}\;{\text{error}}\;{\text{rate}} = {\text{Square}}\;{\text{root}}\;{\text{of}}\;\left[ {\left( {0.{3}} \right)^{{2}} + \left( {0.{7}} \right)^{{2}} + \left( {0.{4}} \right)^{{2}} + \left( {0.{1}} \right)^{{2}} + \left( {0.{2}} \right)^{{2}} + \left( {0.{2}} \right)^{{2}} + \left( {0.{3}} \right)^{{2}} } \right]^{{{1}/{2}}}$$

**Table 4:** Faults and uncertainties in all tools used for measuring quantities.

Quantities	Tool	Levels	Error rate
Ambient temperature	Fluke meter	0–99.9 °C	± 0.3
Climate wind speed	Fluke meter	0–14.9 km/h	± 0.7
Ambient humidity	Fluke meter	0–99.9%	± 0.4
Reflected temperature	Drone-Camera	0–60 °C	± 0.1

Calculated results			Uncertainty
Osama variable 1	Drone-Camera	0–1	± 0.2%
Osama variable 2	Drone-Camera	0–1	± 0.2%
R-value	Drone-Camera	–	± 0.3%

The entire error rate =  ± 1.02%

The entire error rate estimated for experimental setup is ± 1.02%, thus being in a normal array.

## Experimental setup

This particular section explains the procedure applied to obtain the basic data required to compute the R–value from the drone and infrared camera setup. The section also highlights how accuracy is dependent on the atmospheric conditions around the drone in order to generate an unbiased data. The basic criterion behind data collection is stability of the drone in order to generate maximum number of photos of the required wall. Also, care should be maintained to only limit number of photos to 10 per wall since the storage on the drone is limited. The drone after attaining a particular height captures photos through thermal imaging and then further estimates the heat losses through each wall. Drones are probably applied in tall or high rise building where reach of human may become a tedious job. The following assumptions are considered before analysing the investigational datasets as prearranged below:

- Normal climatic conditions were observed before drone flight being shower likelihood, wind velocity, dry bulb temperature and ambient humidity. The moistness fluctuated among 20–80%, wind velocity about 15 km/h, and DBT fluctuated amid 2–41 °C.
- Also drone balancing and height difference constraints are considered in drone guide for proper flight.
- Thermal images were taken once the drone reaches it requisite height. An early recording hampers the battery life of the drone. A long battery life is required in order to complete the operation for all major walls.
- The data captured was advanced to the model framework for more calculations within the R-value for each wall.

The total time taken to complete the data collection process is approximately 20 h (4 h per day) for different times throughout the day to attain uniformity in results. The total time duration was two months so as to cover most of the climatic conditions in a month as shown in Table 5. More detailed explanation of test runs is provided in Supplementary Sect. A3.

**Table 5:** Test-run details for a drone setup.

Day	Location	Time	Weather conditions	Flight time (h)
1	New Delhi	10:00 AM–4:00 PM	Sunny	4
19	New Delhi	10:00 AM–4:00 PM	Cloudy	4
24	New Delhi	10:00 AM–4:00 PM	Rainy	4
47	New Delhi	10:00 AM–4:00 PM	Windy	4
60	New Delhi	10:00 AM–4:00 PM	Cloudy	4

## Results and discussion

Drones offer several advantages over traditional methods of building inspection when it comes to detecting and assessing building deterioration. One major advantage of drones is their ability to access hard-to-reach areas, such as roofs and facades, without the need for scaffolding or other costly equipment. This allows for more thorough inspections and can help identify problems that might otherwise be missed. Additionally, drones equipped with high-resolution cameras and other sensing technologies can provide detailed imagery and data that can be used to identify structural issues, moisture intrusion, and other signs of deterioration. Finally, drones can be operated remotely, reducing the need for human workers to be in potentially hazardous situations. Overall, drones offer a safer, more efficient, and more effective method for building inspection and maintenance, making them an increasingly popular choice for building owners and managers.

The data collection process was widely based on a simple technique of flying the drone to a specific height where R-value needs to be estimated. Furthermore, pictures were taken at a particular distance not too close and not too far. A rapid data obtaining process was employed which was based on 3 simple procedures; direct, concentrate, and capture the thermal image. The specific segment elucidates the importance of detecting the building deficiencies while designing the insulating materials requirements for building envelopes. The values were attained with supreme care, with special attention on furnishing an appropriate process of quantifying the route, absorption, updraft clarity, current span, and distance. The process needs to be performed quite quickly due to which the nature of the substances may not be taken into consideration. The values are later adjusted based on the values present in the software.

### Influence of ambient temperature on variables

**Figure 5:**
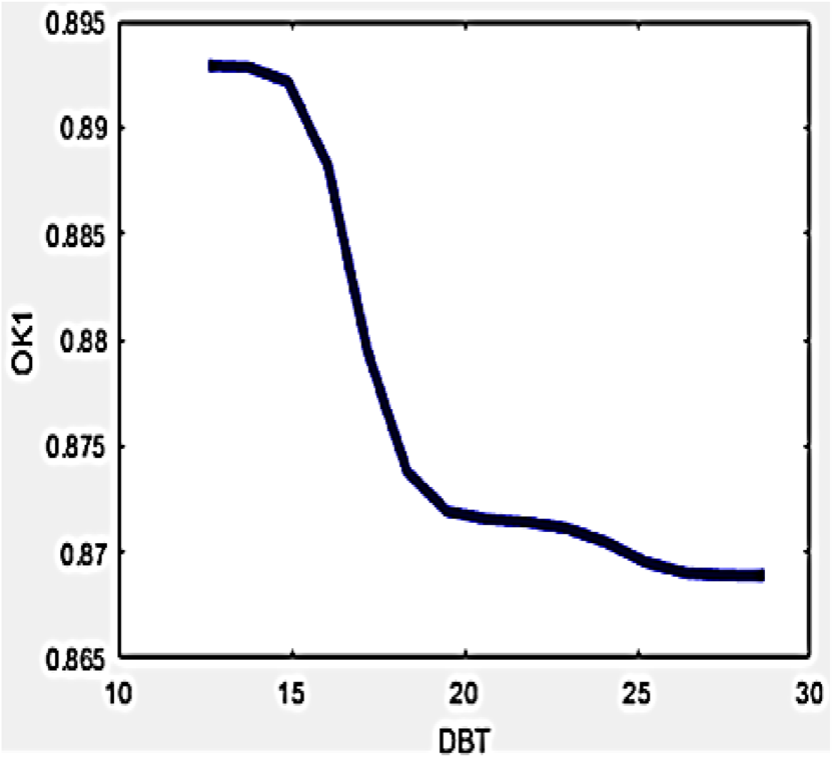
Variation of O_k1_ with DBT.

**Figure 6:**
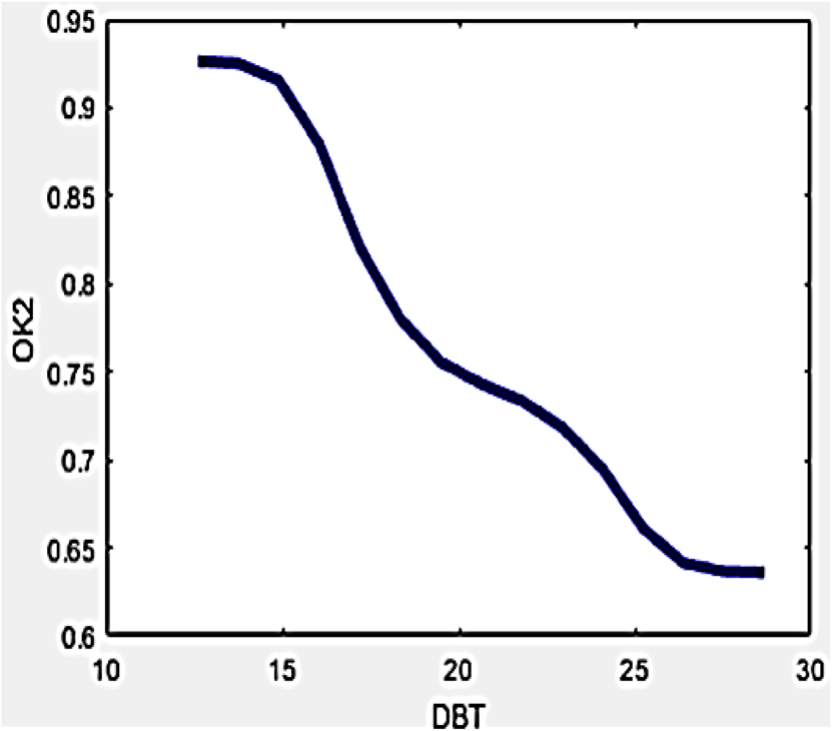
Variation of O_k2_ with DBT.

In drone detection process, ambient temperature is an essential operational parameter which considerably influences the temperature measurement process, thereby might be modified by means of hybrid formula. Frequently high levels of ambient temperatures may furnish lots of barrier for correct temperature measurements in the wall area since the draft of hot air present in the front of camera might pertain to higher temperature. On the contrary, occurrence of breeze delivers an inferior reading within the camera. Thus, existence of positive draft brings uncertainties in the measured values which can be removed by considering the variables O_k1_ and O_k2_ which environment uncertainties into consideration while estimating the amount of degradation in any wall. The inter-relationship among mutual variables can be understood with Figs. 5 and 6.

### Influence of ambient humidity on variables

**Figure 7:**
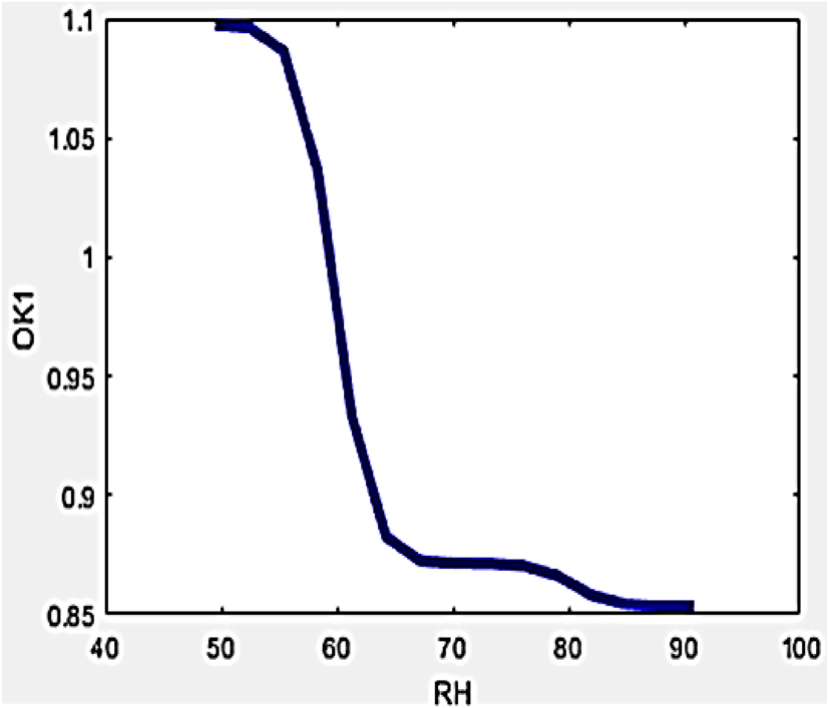
Variation of O_k1_ with RH.

**Figure 8:**
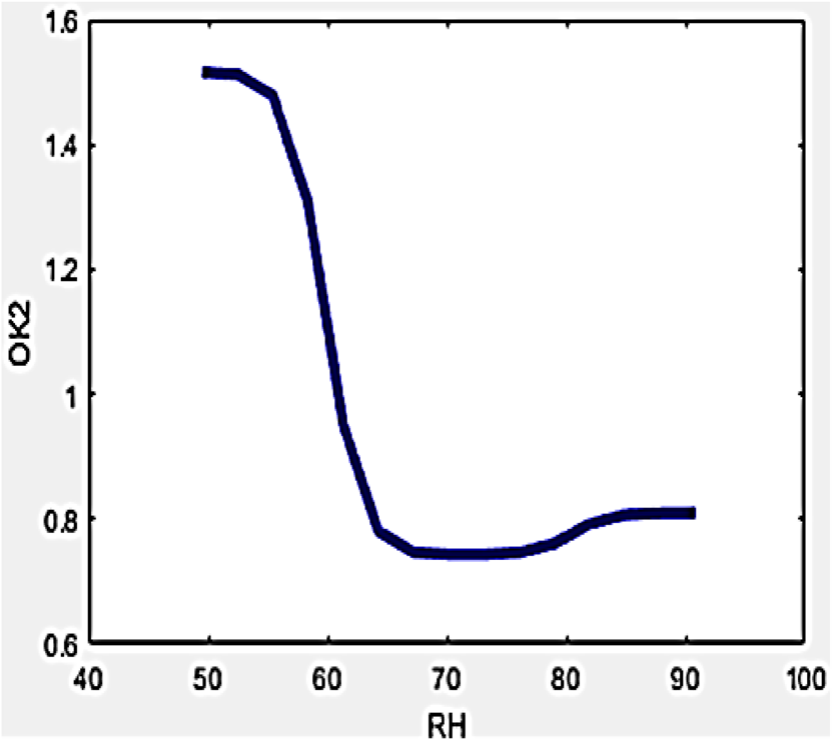
Variation of O_k2_ with RH.

Differences in ambient humidity is estimated for buildings operational conditions with the usage of a drone-camera integration shows a prominent part in furnishing thermal resistance. Occurrence of higher levels of moisture amount in climates results in uncertainties within the drone-camera measuring process due to the droplet formation on the surface of aluminium and black tape. Additionally, a coating of moist water droplets accumulates over the wall structure wherever reflected temperature is to be measured. These inherent errors can be nullified or avoided by incorporating developed variables in the formula developed for evaluating the R-value. The relationship of the developed variables with RH can be interpreted by Figs. 7 and 8 displayed below.

### Influence of ambient wind speed on variables

**Figure 9:**
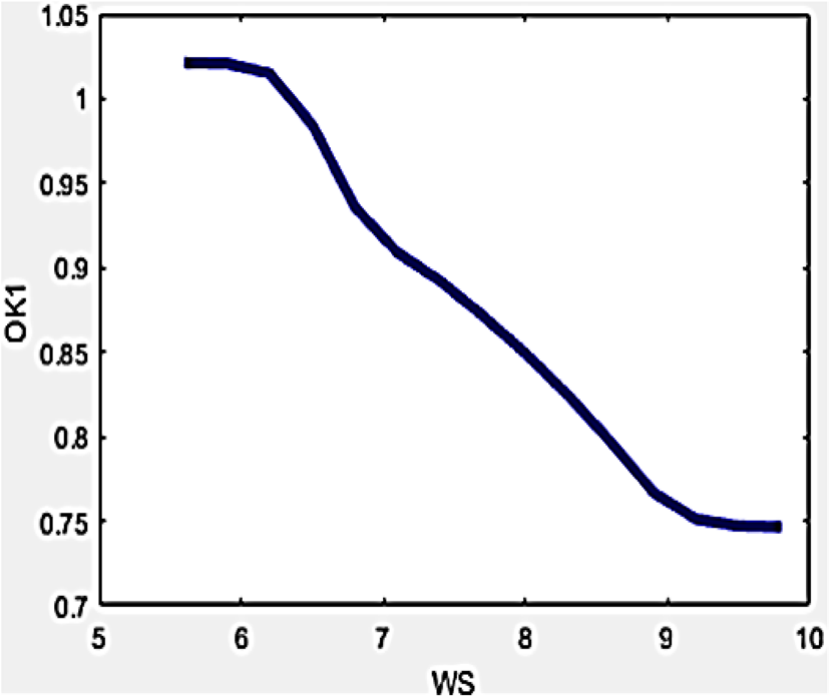
Variation of O_k1_ with WS.

**Figure 10:**
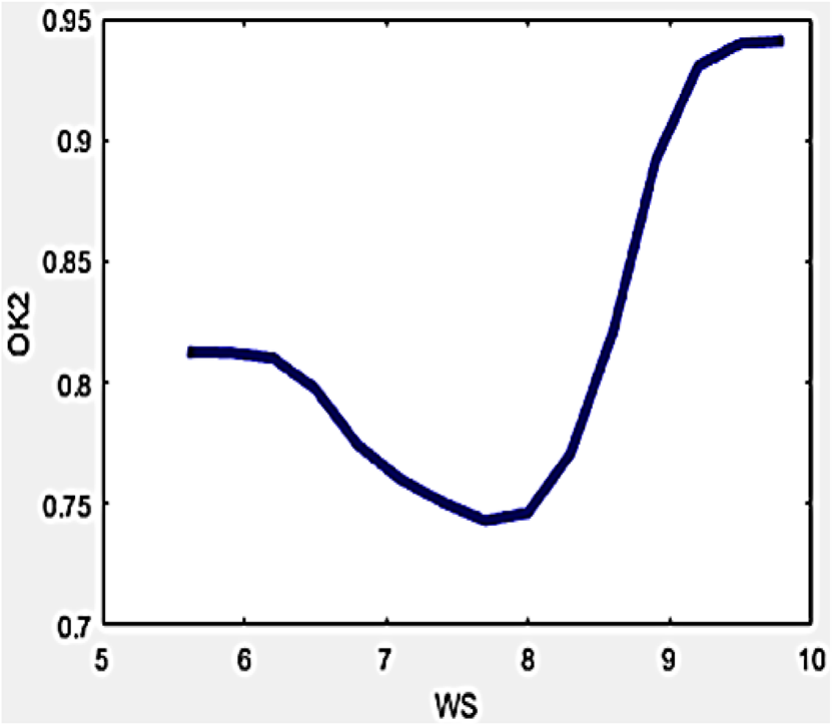
Variation of O_k2_ with WS.

Drone stability is an indispensable constraint during the measurement of the R-value in reflected temperature approximation. Higher levels of ambient wind achieved pertains to imbalance in the drone which becomes unstable yielding errors in temperature measurement. Even though regular standards are maintained in the entire investigation, while it might necessitate a drone to be positioned while directing the camera towards the measurement stamp. These inherent errors can be nullified or avoided by incorporating developed variables in the formula developed for evaluating the R-value. The relationship of the developed variables with wind speed can be interpreted by Figs. 9 and 10.

### Prediction and validation of Osama formula with artificial intelligence (ANFIS)

In this section, the prognostic capability of ANFIS model is estimated to present a rational correlation amongst R-value and climatical situations, comprising ambient temperature, ambient draft air, and relative humidity in this research. Investigational outcomes for numerous walls were attained beneath diverse climatical influences.

From given involvement and influences contribute to a significantly extensive collection of data that provides massive investigational outcomes that require period, effort, power, and oil. This study proposes an AI method (ANFIS) that has been adopted to permit an applied and steadfast response forecast with negligeable information input inexactness, comprising of a partial numbers as inputs. Different datasets generated from the investigated drone data, training and validation obtained via RSM is examined by means of the Sugeno-type fuzzy inference system, which mainly purposes on a sophisticated process that engages the regression analysis demonstrating and the inclined descent technique of the back-propagation^41^.

Soft computing is carried out to enhance the enormous functionality of the assembly established for the difficult drone settings. Previous revisions have furnished the efficiency of ANFIS plannings consisting of 3 training datasets and 4 primary levels which needs to be actuated in the system for diverse type of situations as shown in Table 6. This is shown in Supplementary Sect. A2.

**Table 6:** Developed level of uncertainty in integrated modelling for available variable function.

Association function	Ok_1_ uncertainty%	Ok_2_ uncertainty	R-value
Triangular member function	0.000013	0.00107803	0.0001
Trapezoidal member function	0.007145	0.00545253	0.03119
Cubic member function	0.000041	0.000703381	0.00001
Gaussian 1-member function	0.000026	0.000366849	0.000007
Gaussian 2-member function	0.000008	0.000375703	0.00003
Polynomial member function	0.007740	0.00945187	0.02604
Generalized Bell member function	0.000009	0.000620366	0.00009
Sigmoidal member function	0.000009	0.000620358	0.00009

**Figure 11:**
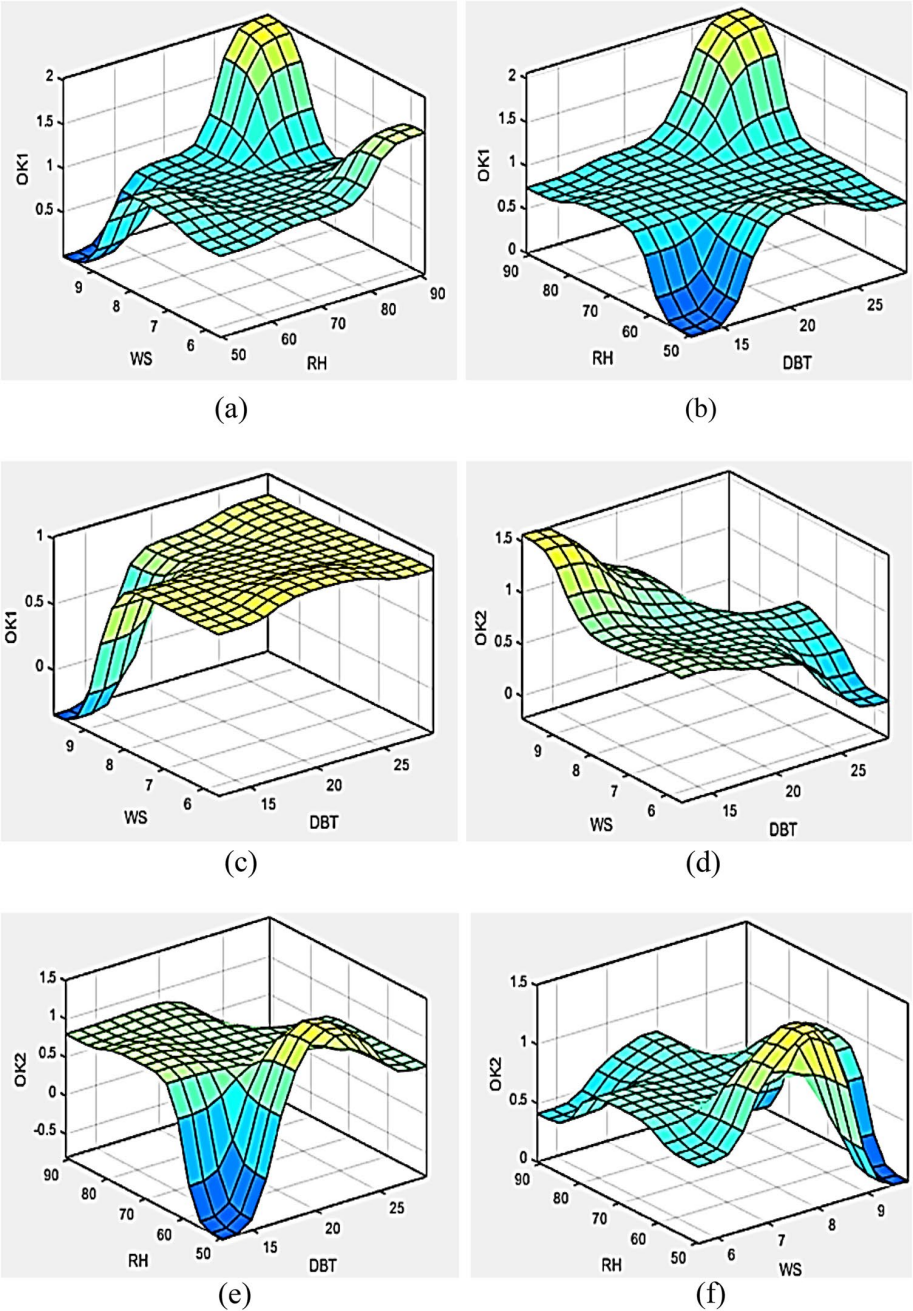
(**a**–**f**) 3-D plots between environmental parameter’s (DBT, RH and WS) and formula constants (O_k1_ and O_k2_).

In the event of developing a feasible framework for available data structure, the claims assured by ANFIS modelling are integrated with environmental factors where they produce its operational constraints based on present variables and acquired input datasets. These algorithms facilitate a viable inter-relationship development for numerous variables and their outcomes associated to ambient environment for the test building as displayed in Fig. 11a–f exhibiting many graphs amongst the natural habitat and formula variables. Mean square nonconformity and percentage of the variance estimation were assessed for all members of the available framework and web topographic anatomy fashioned in Sugeno ensuing datasets in ANFIS modelling. For various kinds of membership purposes, fault proportion is attained so as to anticipate the ideal working algorithm amongst all. Smallest error is accomplished for gaussian function 2 for O_k1_ variables whereas gaussian function 1 membership function attained lowest error rate in O_k2_ variables of the Osama formula. Also, gaussian membership 1 predicted better outputs for thermal resistance R-value as evident from Table 6.

The inherent uncertainties in all developed model for various membership functions are tabulated in Table 6 with their respective estimation of lowest error rates emphasized with yellow markings. Preparing efficient ANFIS models, yield dependable data that is quite identical to the prior hypothetical information furnished handout to train and examine the outcomes of the study. Also, prognostic values inspected for ANFIS classes recognizes inter-relationship between each variable demonstrates the authentication of the projected formulation as the outcomes accomplished are fairly adjacent to those achieved in the formulation.

The outcomes estimated by hybrid models (ANFIS, conventional theoretical and novel theoretical) were evaluated on the basis of regression formulas such as root mean square error (RMSE) and fraction of variance R^2^. Often these statistical tools are employed to estimate the deviation between the experimental and predicted responses. Fraction of variance (R^2^) works on the concept of linear regression taking in account all the over and under estimations within the system. RMSE is employed to estimate how close the residuals (experimental data and forecasted data) are to the best fit line. To validate and cross verify the building parameters outcomes, these were tested for different uncertainties using statistical tools. Different types of regression analysis were performed to evaluate the feasibility of these soft computed hybrid models. The accuracy of the predictive model was validated by considering the regression formulas like RMSE and R^2^. Previous prediction models were deemed accurate if RMSE evaluated was close to zero. Conversely, the fraction of variance of the forecasted data should be close to 1 for accurate fitting model. In the present research, all implemented models complied with the above statistical error standards facilitating a reliable and consistent forecast.

### Optimisation of constants with the help of response optimizer

**Figure 12:**
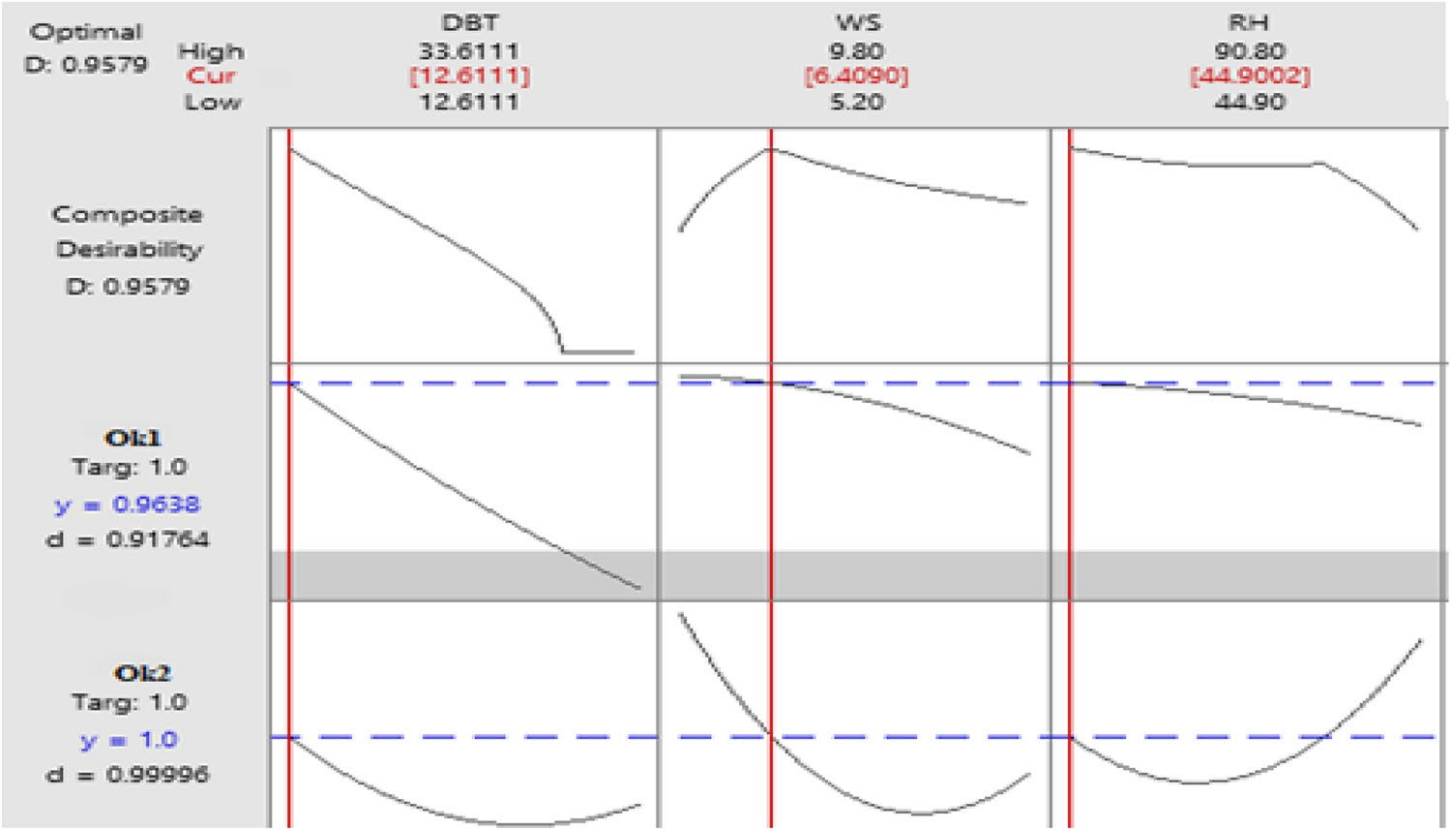
Pareto-optimal conditions attained with response surface optimizer.

Multi-objective response optimization (MORO) is furnished with the aid of Minitab 18 and the outcomes are displayed together in Fig. 12. In accordance with the graphical representation of multiple response optimization, the user requires highest O_k1_ and O_k2_ somewhere near 1. The predicted optimum values for O_k1_ and O_k2_ are concurrently achieved at an optimum grouping of 44.90% relative humidity (RH), 12.61 °C dry bulb temperature (DBT) and 5.20 km/h wind speed (WS).

### Validation of environmental parameters as inputs by ANOVA approach

Constants of the formula O_k1_ and O_k2_ are found to be 0.91 and 0.99 with the aid of the regression approach. The values of R^2^ and R^2^ (adj) for O_k1_ and O_k2_ calculated and found to be 98.65% and 98.35% respectively. Henceforth, the implicated model parameters are reasonably enough for present established results. Simultaneous analysis with the help of p-value test, F-ratio test, and variance (ANOVA), are executed and the results are displayed in Table 7. Among the required output values, linear properties were found to be 0.01 which might be reckoned as substantial. F-value of the lack-of-fit has also been estimated for output parameters with their respective values as displayed in Table 7.

**Table 7:** ANOVA for the RSM models.

Output responses	Method	Regression	Square	Fit
O_k1_	F-value	0.44	2.43	0.6913
	p-value	0.88	0.107	–
O_k2_	F-value	0.51	0.44	0.8194
	p-value	1.88	0.51	–
R-value	F-value	1.22	2.43	0.9014
	p-value	0.88	0.11	–

### Validation of the proposed formula

In previous section, Osama formula reported reduced error rate (RMSE) in comparison to earlier theoretical model which did not take in consideration the environmental variations in air^42,43^. Furthermore, zenith R^2^ value approaching 1 was achieved in the very same integrated formula. Table 8 shows the comparisons for the observed and fitted response values. The computed percentage variation between fitted and actual model is also established, thereby showing values that indicate the impact of the variables on the outcomes are provided in each final row. The simulation developed via regression analysis are displayed in Fig. 8.

**Table 8:** Comparisons for the observed and fitted response values (confirmation tests).

Environmental Inputs fed into the system at 12.61 °C DBT, 6.40 km/h WS and 44.90% RH			
Output response	Fitted	Actual	Percentage error (%)
O_k1_	0.9238	0.91764	0.66
O_k2_	1.00	0.99996	0.04
R-value	0.634	0.626	1.27

To validate and justify the selection of Osama formula, previous model was recollected and compared with the experimental model as presented in Table 9. Data comparison was established on experimental survey for different walls outcomes. The present model has shown the same pattern of higher accuracy (lowered RMSE with higher R^2^) in Osama formula as it takes environmental variations in consideration, thereby validating the proposed formula. Validation of the present model with earlier models developed by AI in similar problems are displayed in Table 10 for comparative analysis.

**Table 9:** Comparison of prediction capability of conventional theoretical models and Osama Formula in accordance with original experimental values.

Model Name	RMSE	R^2^
Theoretical	2.470	0.68
Osama formula	0.547	0.97

**Table 10:** Comparison of accuracy of various models.

References	Model	RMSE	R^2^
Seraj et al.^44^	ANFIS-GA	3.470	0.38
Khan et al.^45^	ANFIS	6.426	0.24
Aghbashlo et al.^46^	ANFIS-ALFIMO	0.423	0.92
Current study	ANFIS-RSM	0.547	0.97

## Conclusions

The modern investigation discovered the possibility of employing a drone-camera arrangement to achieve green building concept which comprised of numerous climatic variables such as dry bulb temperature, wind speed, and relative humidity. Also, a novel formula, namely Osama formula, was developed which was employed to estimate the thermal resistance of the walls and finally total heat loss while accounting changes in environment. The investigational outcomes were generated and established a comparative study to those obtained from the formula. The conventionally generated dataset would have consumed lots of time with the complexity also involved with the problem which would be difficult in the whole process as the environment keeps on varying. Therefore, the exemplary analysis discovers and approves the higher prognostic accurateness of building resistance walls by integrating the discrepancies in climatic variables which vary the attained data. Additionally, the novel formula generated optimum probable outcomes nearer to real time attained values. Foremost outcomes of the analysis have been explained below:

- The research addresses the primary concern of achieving green buildings with minimum power usage which has been reduced by analysing the deterioration of buildings for numerous climatic variations.
- The research also aids in evaluating the primary heat-based losses through building envelope components which hamper green building concept. Appropriate amendments in building elements like insulation over these might lower the power transfusion rate.
- Also, the analysis aids in pin pointing the particular position of the discrepancy, thereby giving a chance to improve the existing design. Further, this location can be improved by insulating or entirely substituting the envelope location. The locations were detected by integrated thermal imaging camera which evaluated the R-value for various envelopes.
- The research found that the R-value degraded over the years in comparison to original value which was earlier in accordance with ASHRAE standards. The obtained R-value led us to valuable computations which explained various modification in designs thereby lowering the energy requirements and increasing the cost effectiveness of the building.
- Existing investigation might help the upcoming inspectors to forecast and assess the R-value for various tall structured buildings by a simple and cost-effective means where energy inspection might not be possible by employing cranes, which is a costly affair, thus enhancing the energy efficiency of the structure.

However, the extent to which the results can be similar if the same procedure is repeated on different buildings would depend on the similarity of the buildings in terms of their construction materials, insulation, and orientation, as well as the external environmental conditions during the measurement. Overall, while drone-based thermal resistance measurements have the potential to provide valuable insights into a building's energy performance, it is important to consider the limitations of the method and account for the potential sources of error that can impact the accuracy and reproducibility of the results. The future scope of this study could involve developing methods to mitigate the impact of indoor conditions on drone-based thermal resistance measurements, such as using sensors to monitor the indoor environment during the measurement process. Additionally, the study could be expanded to include a larger sample of buildings to assess the generalizability of the findings.

## Supplementary Information

Supplementary Information.

## Data Availability

The datasets used and/or analysed during the current study available from the corresponding author on reasonable request.

## Abbreviations

AI: Artificial intelligence
SH: Specific humidity
WF: Working fluid
IoT: Internet of things
ANFIS: Adaptive neuro-fuzzy inference system
IRT: Infrared thermography
MT: Mean temperature
AC: Air conditioning
OK: Osama Khan formula
NBC: National building code
DBT: Dry bulb temperature
SH: Sensible heat
WV: Wind velocity
LH: Latent heat
WBT: Wet bulb temperature
RSM: Response surface methodology
RH: Relative humidity
RMSE: Root square mean error
R^2^: Fraction of variance
AT: Average temperature
SC: Shading coefficient
O_K1_: Osama khan variable 1
O_K2_: Osama Khan variable 2
