# Supplementary material for: Application of artificial intelligence in green building concept for energy auditing using drone technology under different environmental conditions
Source: Sci Rep. 2023 May 21;13:8200. doi: 10.1038/s41598-023-35245-x (PMC10200798; doi:10.1038/s41598-023-35245-x)
Supplement: Supplementary file 1 — Supplementary Information. [file 41598_2023_35245_MOESM1_ESM.docx]

**Supplementary Materials**

**A.1 Response surface methodology in drone estimation**

Vast domain has successfully implied RSM technique to furnish prognostic values with a faster and efficient way, while also simulating the problem according to its requirement. The tool also optimizes the responses based on the set of available parameters. Henceforth, RSM is often employed for performing simulations, optimization and vary levels of any number of inputs for a specific dataset. The investigated datasets are perceived with response surface regression method polynomial modelling of the second-order which were built by means of Equation (24):

$Y=\beta_{o}+\sum_{i=1}^{k} 1.\beta_{i}X_{i}+\sum_{i=1}^{k} 2.\beta_{ii}X_{i}^{2}+\sum_{j\geq i}^{k} 3.\beta_{ij}X_{i}X_{j}+ \varepsilon$………………………………(24)

where Y is the required outcome, X_i_ are numeric values of the factors, whereas terms β_0_, β_i_ , β_ii_ and β_ij_ are regression coefficients, i and j are linear and quadratic coefficients, and ε is the experimental error. Response surface graphs were drawn with the aid of these fitted representations.


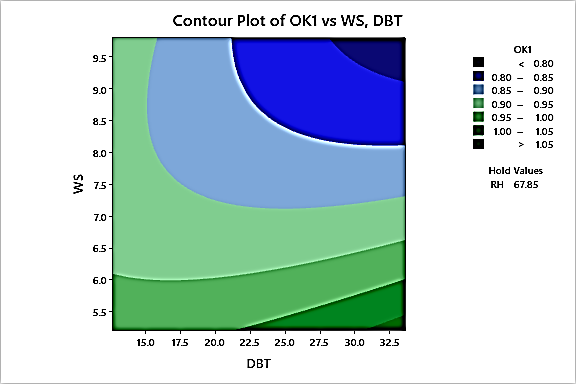

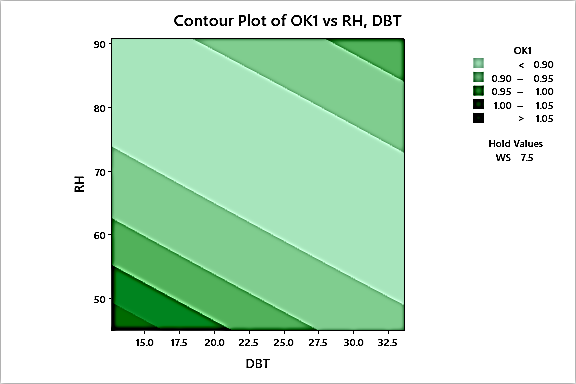


**Figure 16:** Contour plot between O_k1_ and DBT-WS **Figure 17:** Contour plot between O_k1_ and DBT-RH


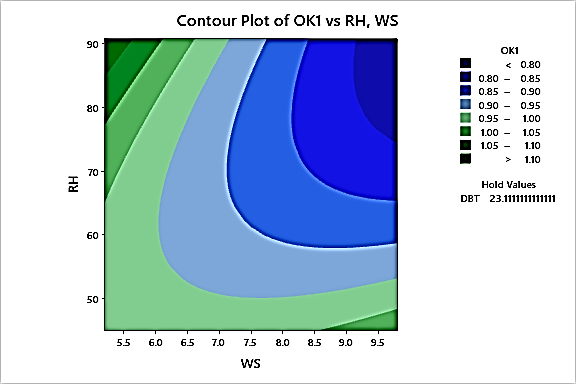

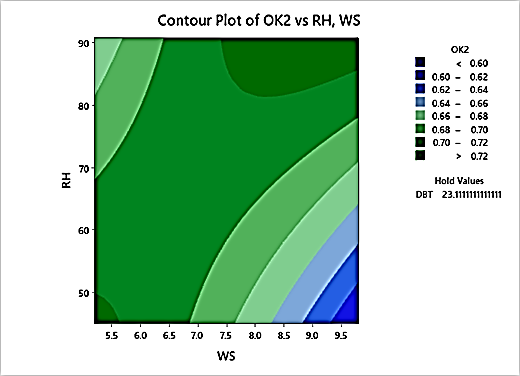


**Figure 18:** Contour plot between O_k1_ and RH-WS **Figure 19:** Contour plot between O_k2_ and WS-RH


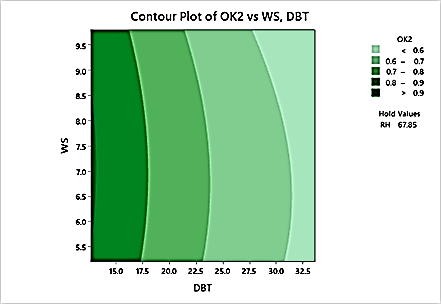

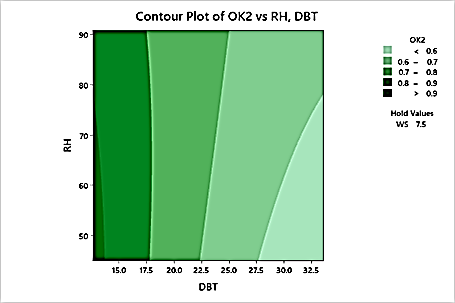
**Figure 20:** Contour plot between O_k2_ and DBT-WS **Figure 21:** Contour plot between O_k2_ and DBT-RH

Drone-camera thermal imaging experiments were performed and the corresponding dry bulb ambient temperature, available wind draft, and ambient relative humidity were employed as the operating variables. Besides, involvement of climatic conditions is not only a complex procedure nonetheless correspondingly it embraces a firm level of error connecting non-linear connection among the developed values, a second order prototype was established to validate the inter-relationship among the climatic constraints and requisite results. The equivalences established are shown in Figures 24 and 25, which establish the inter-relationship amongst many climatic parameters and variables

O_k1_ =1.2718 - 0.000480 DBT - 0.05013 WS + 0.7698 RH………………….(24)

O_k2_ =1.0753 - 0.016093 DBT + 0.00183 WS +0.489 RH…………………....(25)

The ideal grouping of environmental parameters of DBT, RH, and WS can be finally attained with the aid of desirability method of RSM. Previous research have also employed this particular approach for validation and significance of the applied [34, 35]. The several outcomes achieved with the aid of desirability technique are then validated by performing confirmatory experimental runs built on the set of optimization condition.

The requirement of this examination is to maximize these two variables (O_k1_ and O_k2_) and attains a value close to the vicinity of one. Initial cluster of designs are shown for O_k1_ in Figure 22 where basically it is a convention of virtual outcomes obtained through employing RSM. Primarily the histogram established prognosticates the variables value to be fairly adjacent to the formula generated standards, afterward displaying lowest error value. Concurrently, Figure 23 exhibits uncertainty level recognized for O_k2_ and includes the variables for these replication outcomes. This also is found to be extremely small and is very adjacently aligned along the centre line of developed bar values of histogram, which is pertained to be an advantageous aspect.


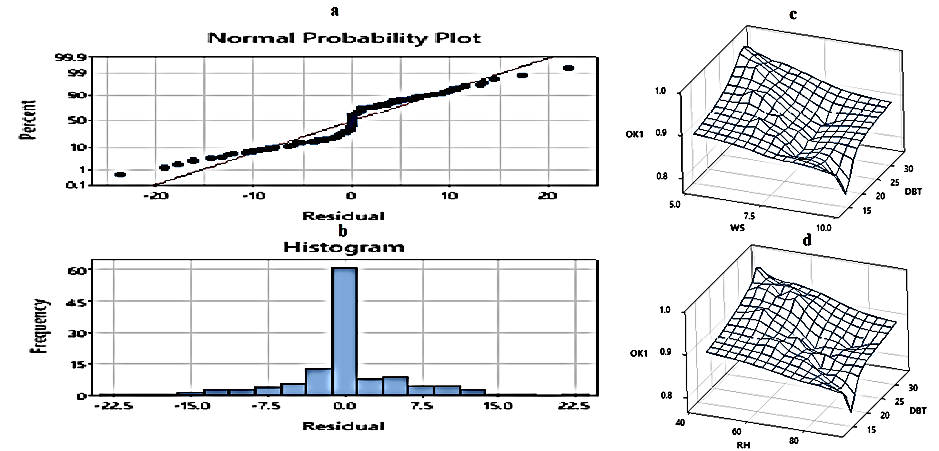


**Figure 22:** Simulation results of O_k1_ with RSM approach


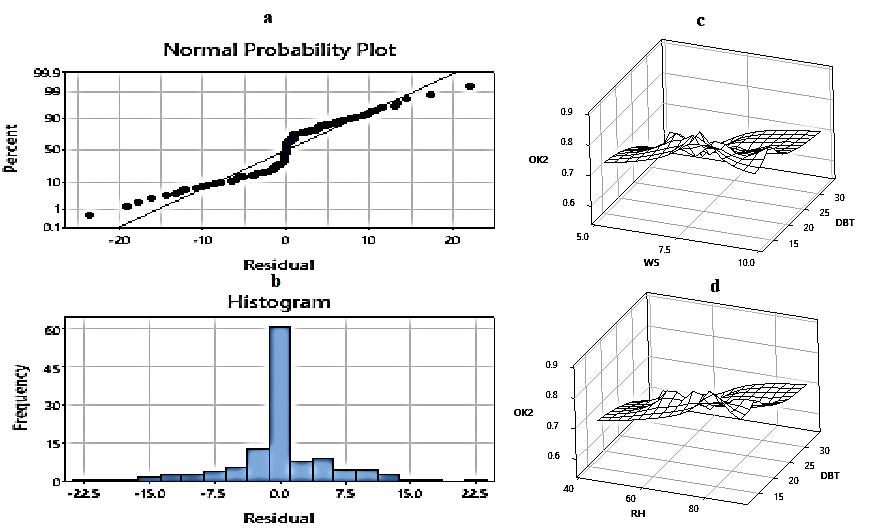


**Figure 23:** Simulation results of O_k2_ with RSM approach

**A2: Rules established in ANFIS**

The specific outcomes have their individually developed models capable of analysing the data and producing a valid framework. This framework established twenty-seven various outcome rules when combining with the investigational climatic parameters. Above rubrics are recognized for couple of variables earlier established in the study known as O_k1_ and O_k2_ are represented in Figure 11 and Figure 14.


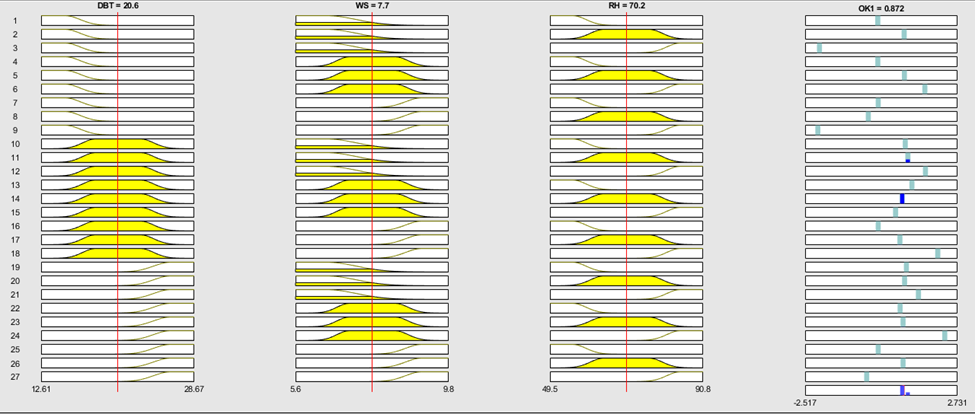
**Figure :** Rules accomplished in developing O_k1_ for different environmental conditions


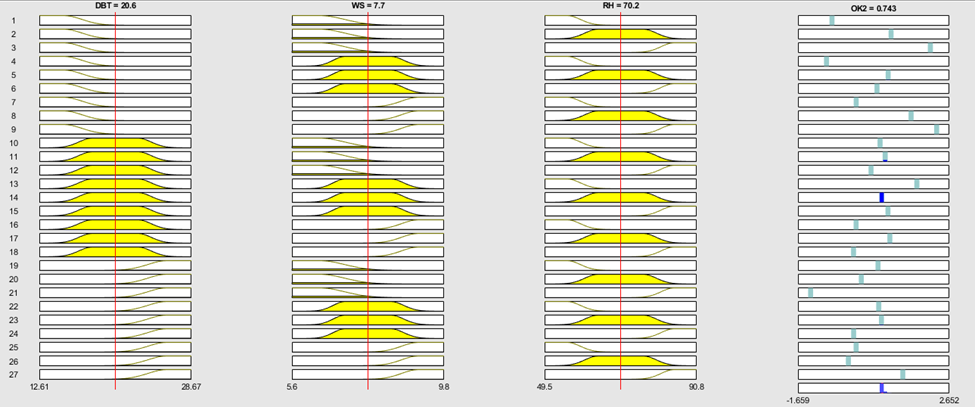
**Figure :** Rules accomplished in developing O_k2_ for different environmental conditions

**A3 Experimental Test Data**

| S.No. | DBT (℃) | Wind speed  (km/h) | Relative  Humidity (%) | Heat Convection Constant (W/m^2^K) | O_K1_ | O_K2_ | R-value (°C/W) |
| --- | --- | --- | --- | --- | --- | --- | --- |
| **1** | 12.61 | 8.2 | 79.9 | 3.1 | 0.87 | 0.89 | 1.83 |
| **2** | 13.89 | 7.8 | 78.6 | 2.2 | 0.87 | 0.86 | 0.69 |
| **3** | 17.39 | 7.4 | 75.4 | 1.9 | 0.89 | 0.82 | 0.42 |
| **4** | 18.22 | 6.9 | 63 | 1.8 | 0.92 | 0.81 | 0.69 |
| **5** | 17.28 | 7.5 | 71.5 | 1.9 | 0.89 | 0.82 | 0.54 |
| **6** | 16.83 | 8.3 | 79.1 | 2 | 0.84 | 0.83 | 1.44 |
| **7** | 19.39 | 8.1 | 79.4 | 1.7 | 0.83 | 0.78 | 0.53 |
| **8** | 20.72 | 8.2 | 76.4 | 1.4 | 0.85 | 0.75 | 0.27 |
| **9** | 21.94 | 7.7 | 78.5 | 0.6 | 0.88 | 0.73 | 0.03 |
| **10** | 20.78 | 7.6 | 77.9 | 1.4 | 0.88 | 0.75 | 0.49 |
| **11** | 17.39 | 9.8 | 80 | 1.9 | 0.78 | 0.82 | 0.44 |
| **12** | 19.61 | 7.4 | 74.5 | 1.6 | 0.89 | 0.77 | 0.35 |
| **13** | 21.33 | 7.1 | 67.3 | 1.2 | 0.91 | 0.75 | 0.08 |
| **14** | 19.89 | 8.3 | 78.4 | 1.6 | 0.84 | 0.77 | 0.28 |
| **15** | 23.11 | 8.2 | 76.4 | 1.3 | 0.85 | 0.71 | 0.07 |
| **16** | 24.06 | 8.1 | 71.4 | 1.6 | 0.85 | 0.69 | 0.13 |
| **17** | 25.56 | 7.8 | 72.8 | 1.8 | 0.87 | 0.66 | 0.20 |
| **18** | 21.83 | 7.6 | 71.9 | 0.8 | 0.88 | 0.73 | 0.01 |
| **19** | 25.22 | 6.5 | 55.3 | 1.8 | 0.94 | 0.67 | 1.71 |
| **20** | 23.11 | 6.3 | 54.1 | 1.3 | 0.95 | 0.71 | 0.05 |
| **21** | 28.11 | 5.9 | 49.5 | 2.1 | 0.97 | 0.62 | 3.06 |
| **22** | 24.61 | 6.8 | 60.8 | 1.7 | 0.92 | 0.66 | 0.14 |
| **23** | 28.67 | 5.6 | 52.4 | 2.1 | 0.97 | 0.62 | 2.80 |
| **24** | 31.11 | 5.2 | 44.9 | 2.3 | 0.99 | 0.59 | 1.26 |
| **25** | 28.22 | 5.9 | 60 | 2.1 | 0.97 | 0.63 | 0.80 |
| **26** | 27.17 | 6.2 | 58.6 | 2 | 0.94 | 0.67 | 2.41 |
| **27** | 29.67 | 5.3 | 56.5 | 2.2 | 0.99 | 0.61 | 0.58 |
| **28** | 27.00 | 7.4 | 72.4 | 2 | 0.89 | 0.67 | 1.89 |
| **29** | 31.67 | 6.7 | 60.7 | 2.3 | 0.93 | 0.58 | 0.55 |
| **30** | 27.11 | 8.2 | 75.3 | 2 | 0.85 | 0.67 | 4.65 |
| **31** | 29.22 | 6.8 | 55.9 | 2.1 | 0.92 | 0.62 | 0.32 |
| **32** | 33.61 | 6.2 | 50.9 | 3.3 | 0.94 | 0.56 | 1.96 |
